# The Mediating Role of New Media Engagement in This Digital Age

**DOI:** 10.3389/fpubh.2022.879530

**Published:** 2022-05-02

**Authors:** Zhang Yujie, Megat Al Imran Yasin, Syed Agil Bin Shekh Alsagoff, Ang Lay Hoon

**Affiliations:** ^1^Department of Communication, FBMK University Putra Malaysia, Serdang, Malaysia; ^2^Department of Foreign Languages, FBMK University Putra Malaysia, Serdang, Malaysia

**Keywords:** new media technologies, social networking, digital age, communication technologies, attitude toward social media

## Abstract

New Media Technologies refer to all kinds of web-related technologies like social networking sites, blogs, online social media networking, and other communication technology forms. The primary purpose of this study is to examine the mediating role of new media engagement in this digital age. Furthermore, this study defines the concept of new media, highlights the characteristics of new media technologies, and analyzes the application of new media technologies in this digital age. To meet the objectives stated, the researchers conduct a questionnaire survey to showcase that new media engagement plays a mediating role between the instructions provided to the newsroom in this digital age and the attitude of people toward social media. Results of the study state that the majority of the individuals in this digital era display positive engagement with new media technologies. People in this age believe that social media is safe and less risky for all individuals with regard to communication and interactions.

## Introduction

In recent years, there has been growing interest in investigating the role new media technologies play in diverse areas including business, media, education, mass communication, and so on (1, 2). New media technologies are popularly referred to as Web 2.0 technologies. These technologies use web technologies like online social networking, blogs, wikis, and other social media platforms to communicate with a larger group audience. The last few years have witnessed a steep decline in the use of old media such as magazines, television, newspapers, radio, and other traditional media forms, while the internet has taken over the place of old media rapidly; and for that very reason, old forms of mass communication are becoming extinct with each passing day.

Several studies, for instance Lysak et al. (3), Andrus (4), and Boulos et al. (5) have demonstrated that television took the place of life magazines; emails and telephone took over the telegraph; and so forth. From these instances, it can be understood that old media has been replaced by modern communication techniques. New media communication technologies supplemented the different forms of old media by working in parallel to meet the demands of different organizations. Nowadays, any kind of mass communication is available on the internet and at newsstands (5). Mass communication media allows the readers to comment in real-time on blog sites and online social networking sites. Developments in the arena of new media technology permit individuals to participate in public discussions (6). The whole of mass communication technology has changed with the recent developments in the field of technology. An entirely new shape has been given to the communication process where people began to relate to new media on a personal level unlike the old media forms (7). Many researchers stressed the fact that the concept of virtual identity is growing popular in recent years, as it will help them to understand the role of new media technologies in developing one's own identity in this digital age (7, 8). This will also help the researchers to analyze how people shape their own identity by communicating through online networking platforms. Another interesting fact of new media technology is that it allows users to participate in socially or politically mass discussions. In fact, these technologies provide the user a personal space to comment their opinion on the computer-generated public sphere. New media technologies make it possible for users to generate online content with a carefree attitude.

Preliminary studies on new media technologies primarily focused on limited effects of the modern means of communication (9, 10). In the field of media, for instance, barely a newspaper reporter will be able to understand the significance of blogs; hardly a television producer will be able to know why people moved from network television to online television, and so forth. One of the recent studies on New Media technologies traced the advancements in transforming knowledge in the digital format (11). It was found that users looking for information, irrespective of the place, time, and convenience led to the digital transformation through the mobile-first strategy. Other interesting findings in this field are as follows: (a) young people spend more time on social media platforms to consume information; (b) people began to verify information through the internet; (c) businesses began to promote their brands, products, and services through online social networking platforms and other social media forms; (d) People working in media began using new media to search for a new audience, cross-check the information, promote the coverage of news, and verify the accountability of the news; (e) advertising/marketing channels use new media technology to create branding for their products and services; and (f) academicians also began using different new media technologies for educational purposes (12). Recent studies underline that there is an increase in the percentage of people using various types of new media technologies from 4% in 2008 to 36% in 2018 (13, 14).

For several years, researchers have been focusing on the role of new media technologies in an attempt to understand their impact on the Digital Age. It has been traced that new media technologies build content with the help of the computer-generated public sphere. These technologies are considered an effective tool to maintain engagement in this digital age. Immediacy and cost-effectiveness are the added advantages of such technologies. As an example, one of the recent studies provided evidence of people going live on the Facebook application, which helped them to reach a much larger audience than expected (15). Although there exists no way to measure the engagement of new media, it has been emphasized that the involvement of people, the connectivity aspect between people, and their level of interest in taking participation will help the researcher define and measure the mediating effect of engagement in this digital age. With the help of advanced technologies such as web analytics, organizations from different arenas can monitor the role of people's engagement in those activities filled on new media technologies like online social networking sites, blogs, wikis, and other social media platforms. Counting the number of likes, shares, views, and comments, one can monitor the role of engagement. Processing of healthcare and travel data using machine learning algorithms in place of the traditional healthcare system to identify COVID infected persons (33).

Overall, the aim of this research paper is to investigate the mediating role of new media engagement in this digital age. Furthermore, this study defines the concept of new media, highlights the characteristics of new media technologies, and analyzes the application of new media technologies in this digital age. This study also attempts to provide a framework representing the relationship between new media and engagement in the digital age. As we all know, the twenty-first century is ruled by new media, and therefore its influence on individuals, organizations, disciplines, and so forth is relatively higher than the old media. The present study is structured as given below: Section Introduction provides an introduction to the topic; Section Literature Review examines the existing literature review on the concept of new media, its characteristics, and the impact of new media on individuals, organizations, and other areas. This section also examines the application of new media technologies in various disciplines. Section Methodology presents the methodology adopted for the study. Section Data Analysis discusses the findings and results of the study. Lastly, Section Results and Discussion discusses the conclusions derived from the study.

## Literature Review

### Understanding New Media

New Media is a new term that is universally applied in multiple ways. Seminal contributions have been made by past researchers on the concept of new media and its contextual use, with regard to communication and technology (7, 10, 16). These studies observed that communication practices, the technology or medium through which the message is communicated, and the social context in which the message is passed are quite important compared to other factors. That is, the message, channel, and source are important to communicate the information. The majority of the literature focused on the above three aspects of new media for a longer duration, besides other advanced practices and technologies like digitization, collaboration, and telecommunication (7, 10, 17). Few other studies defined new media as a web-related technology that uses advanced computer technologies to transmit messages to a larger group audience (18).

From the term new media technologies, it can be understood that such newly developed technologies use specific computer technologies to process the information and communicate it to the audience (19). A networked telecommunication system is used to interact between the users. Communication in the new media technology occurs when two electronic devices are used to share information supported by advanced technologies and software. Emails, chat rooms, social media services, instant messaging, and so on are the best examples of new media technology communication. These platforms made the communication process easier, resulting in the establishment of a universal networked society. Additional advantages of new media services include allowing the people to work together irrespective of geographical differences, promoting globalization, establish worldwide socio-political dialogues, and framing sociological structures across the disciplines (20). A number of authors have also recognized that communication through new media has changed the whole process of communication. Most importantly, new media is known for convenience and smooth communication processes (21).

Most early studies, as well as current work, stated that the term new media encompasses various aspects like the message, the medium used to transmit the message, the technology used to transmit the message, the time during which the message is transmitted, and the social context in which the message is transmitted (22). Certainly, the term media refers to the technology through which the message is communicated. From the above statements, it is inferred that the medium through which the message is transmitted is more important than the content of the message.

### Characteristics of New Media

This section presents a review of recent literature on the characteristics of new media. The wide range of new media technology characteristics includes—communication, collaboration, convergence, creativity, and community. In general, new media technologies are relatively concerned about one or other forms of communication. Blogs, which are known as one form of new media technology, allow the users to publish content on one's own web pages. Hence, the blog is an alternative web-technology communication method of transmitting information to large group audience. Furthermore, blog posts are not one-way communications, and thus demand the readers to interact/comment/post their reviews/opinions online. Therefore, there is a factor of engagement in the whole communication process, which is two-way and interactive in nature. In short, communication through new media can be categorized as collaborative, networked, and unidirectional (23). In this rapid networking age, the term viral is used to indicate the rapid speed of transmission, for instance, viral video, viral marketing, viral branding, and viral posts. Furthermore, the participants involved in new media technology communication play the role of both sender and receiver. They can even post their opinion/feedback with the support of a computer-mediated communication process.

It was reported in the literature that, if not all new media technology, at least a few of them facilitate collaboration through the internet. Much of the past collaboration happened through emails. Multiple documents could be shared with multiple people at the same time, which saves time (22, 24). Although there were minimal disadvantages of such email collaboration, it was one of the most effective means of new media communication. Such collaboration is available on Wikipedia, an online encyclopedia. Unlike wikis and emails, blogs have limited collaboration and can be shared among a specific group of people. Recent studies found that google docs permit users to share documents with each other (24). It also allows two users to work together on the same document. While social networking platforms like Facebook, Skype, WhatsApp, and others, users collaborate on the aspects of virtual communication and audio conversing. The majority of the social media platforms permit users to virtually connect with each other by means of conference calls and chats (25).

Another interesting characteristic of new media is that it fosters a sense of community. New media technologies like Skype, Facebook, Yahoo groups, Usenet, Webkinz, YouTube, and others incorporate a sense of community among the users by creating logins. The creativity of the users is the next surprising feature of new media technologies (24, 25). No user plays a passive role anymore, instead, they play an active role in transmitting the messages through web technologies. New media technologies enable its users to create videos, edit videos, post blogs, post reviews/comments on blog posts, post reviews for products/services offered by brands both national and international, and finally create content on wikis. People in this digital age not only read, listen to, or view the content posted online, but also edit, comment, and posts feedback. Hence it can be stated that there is a factor of engagement or interactivity within new media technologies. The new media technologies are broadly known for their convergence with media, technology, and other forms of web-related communication technologies. Apart from the above characteristics of new media technology, a few other features include digitized messages, multi-way communication, and a smooth flow of information (26).

### New Media and Engagement

**Figure 1:**
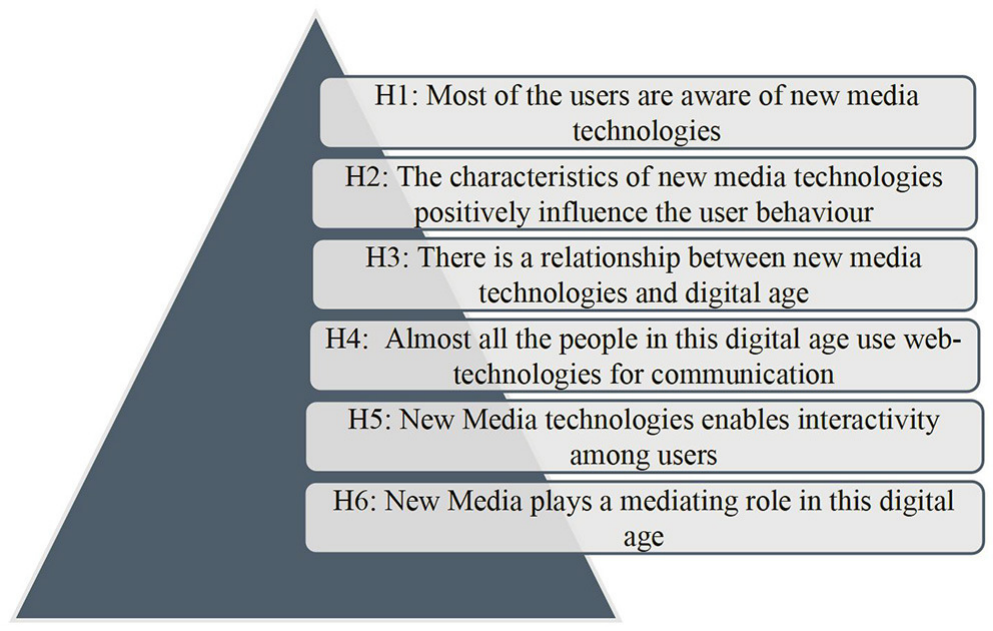
Proposed hypotheses of the study.

Over time, extensive literature has developed on examining the role of engagement in new media technologies. It was traced that new media becomes the most significant channel across the arenas to promote news, education, marketing, branding, and so on. Specifically speaking, people use the new technologies to share critical information with a large group audience. Multiple studies also discussed the use of new media and observed that people tend to spend more time on the newly developed technologies to read, listen, view, comment, and share information with one another (27, 28). These studies also found that user engagement plays an important role in transmitting the message to the target audience. Likewise, recent studies in the field traced that most people in this digital age posts activities through various social media platforms. In short, it can be stated that user participation is much important to process any kind of information (29–32). These new media technologies are changing the behavior of people, enabling them to interact online, create online communities, and become socially engaging. Thus, it can be argued that engagement of the audience/users/individuals is crucial in new media technology communication. Negligible studies have tried to examine the mediating role of new media engagement in this digital age and thus, this paper aims to examine the mediating role of new media engagement in this modern digital age. In the light of the above literature, the study proposes the following hypothesis as represented in Figure 1.

## Methodology

The current study mainly concentrates on exploring the mediating role of new media engagement in the digital age. It also examines the concept of new media, identifies the different types of new media technologies, and the characteristics of new media technologies. The study also examines the application of new media technology in a digital age. The analysis of the current study has been conducted through secondary literature and primary analysis as well. The study has developed hypotheses based on the analysis and review of the secondary literature. The formulated hypotheses are tested using a questionnaire. Thus, the current study gathers all the required data using a questionnaire and hence the study is descriptive. Using the results of the questionare the researcher will evaluate the hypotheses. This method involves describing the objectives, defining the population, selecting the sample, and later interpreting the data and results.

The population is nothing but the complete set of individuals or objects bound together by certain common characteristics. The population is of two types- target and accessible. The target population consists of those for whom the researcher wishes to generalize the findings of the study. While the other set of the population acts as the sub-set of it. In the current study, the total population includes all the people who fall under the age group of 18–50. From the total population, the study derives the required sample. The term sample includes the individuals selected to participate in the study. These selected participants are popularly known as respondents/subjects of research. Sampling methods are of two types—probability/random sampling and non-probability. To carry out the research, this study employs a random sampling method. At times, researchers also tend to use purposive sampling with the aim of obtaining required data from a certain area of research.

**Figure 2:**
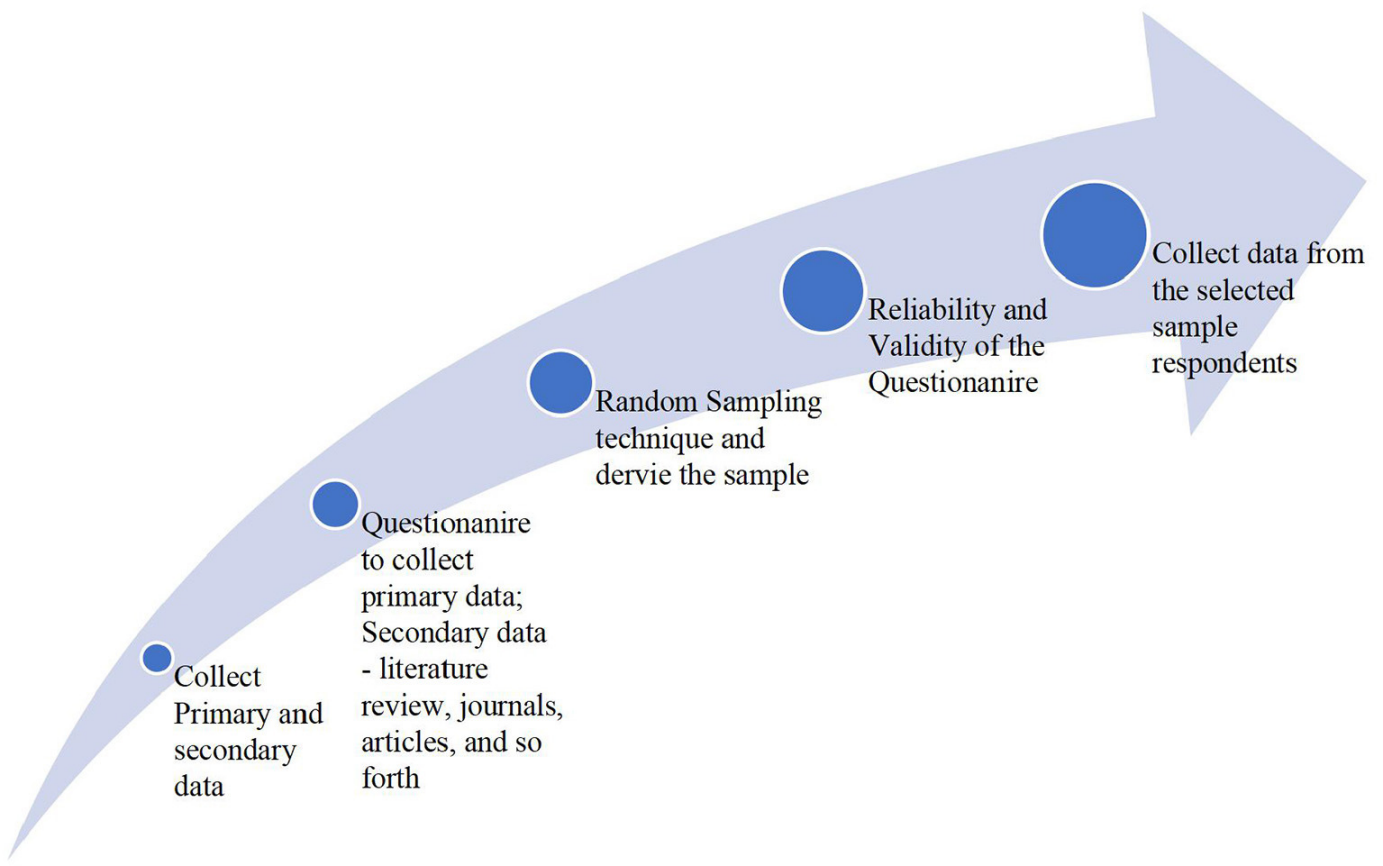
Flow chart of the methodology.

For the purpose of fulfilling the objectives of the study, the study selects a sample consisting of 100 respondents between the age group of 18–50. Both primary and secondary data are collected to find out the results. The primary data is collected using the structured questionnaire designed to collect the required data from the participants. The collected data will help the researcher to examine the mediating role of new media engagement in this digital age; review the attitude or behavior of users who use new media technologies to communicate, and examine the factor of engagement and interactivity among users with a special focus on the digital age. The researcher collects the secondary data required for the study from research articles and other journals. Besides, the study measures the formulated hypothesis with the help of 3-point Likert Scale. A pilot study is conducted to check the reliability and validity of the questionnaire, for 30 respondents. Finally, all gathered information was collected and analyzed to answer the research purpose. Figure 2 presents the methodology followed in the study.

## Data Analysis

**Figure 3:**
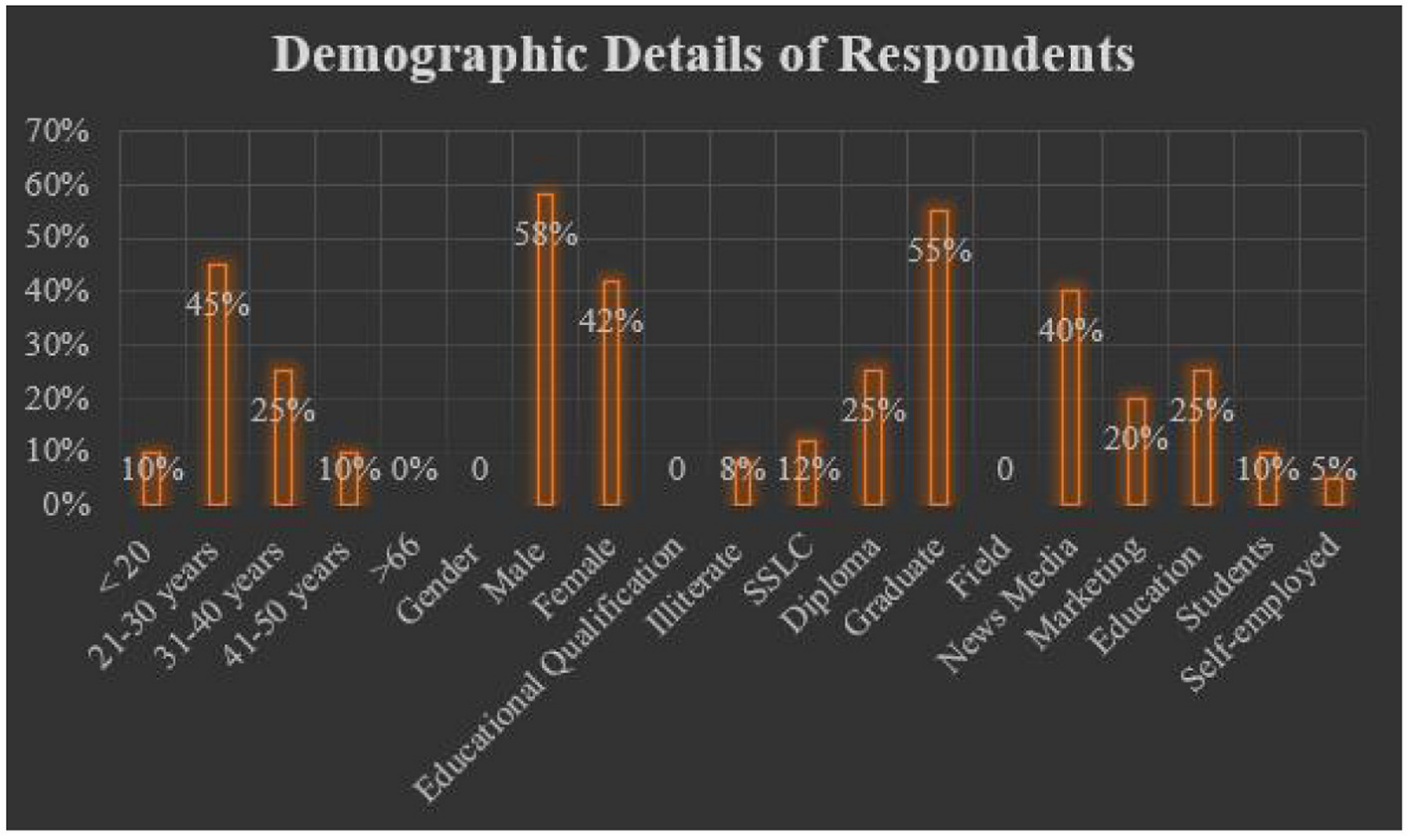
Graphical representation of respondents' details.

This section of the article presents the data gathered from the selected respondents through the questionnaire. Firstly, the demographic details of the respondents are presented, along with the duration, context, and other essential details. The duration of the study lasted for 4 weeks, that is from the end of November to December. To all these 100 respondents, the questionnaire was distributed through online sites. All the participants attended the questionnaire with extreme dedication, as they were asked to fill the questionnaire in an appropriate manner. Although the questionnaire was distributed to 150 participants that include young people, employees working in various organizations like marketing, media, press, education, and self-employed; only 130 were received of which 100 were valid. These 100 valid responses were used for further analysis. Table 1 records the demographic details of the active respondents. Figure 3 represents the graphical representation of demographic details.

**Table 1:** Demographic details of the participants.

	**Percentage of respondents (%)**
**Age**	
<20	10
21–30	45
31–40	25
41–50	10
>66	0
**Gender**	
Male	58
Female	42
**Educational qualification**	
Illiterate	8
SSLC	12
Diploma	25
Graduate	55
**Field**	
News media	40
Marketing	20
Education	25
Students	10
Self-employed	5

From Table 1, the following deductions can be recorded: (1) Most of the respondents (45%) fall under the age group of 21–30; (2) 25% of the respondents fall under the age group of 31–40; (3) 10% of the respondents fall under the age group of 41–50; (4) 10% of the respondents fall under the age group <20. Of all the active respondents, 58% of them were men and 42% of them were women, who were working in different organizations associated with brands, marketing, and so on. The majority of the respondents work in news media followed by education, marketing, students, and self-employed individuals, respectively. The statistics given in Table 1 clearly state that almost all the participants either work in a large organization or are entrusted with new media technologies in this new digital age.

Table 2 presents the responses to the questionnaire collected from the participants of the study.

**Table 2:** Responses of the participants.

**Distribution of the questionnaire**	**Number of respondents**	**Percentage (%)**
Total participants	150	100
Fully completed	130	86.6
Valid questionnaires	100	66.6
Total respondents considered for this study	100	

From the statistic given in Table 2, it can be deduced that the questionnaire was distributed to 150 people that including employees working in marketing, press, media and others, students, and self-employed individuals. Of all 150, 130 questionnaires were completed and 20 of them were incomplete. Of these 130 questionnaires, only 100 were considered valid and reliable.

## Results and Discussion

This section of the study presents the analysis of the data gathered through the questionnaires. The data analyzed with the help of the questionnaire are presented in Tables 3–**6** followed by a discussion of the results. An opinion poll is used to collect the data for the study. All the participants filled out the questionnaire and submitted their results online. These opinions submitted by the participants are analyzed using a 3-point Likert scale. Table 3 records the details of how many people understand new media technology.

**Table 3:** Concept of new media technology.

**About new media technology**	**Respondents' responses in percentage (%)**
Partially aware of new media technology	30
Unaware of new media technology	15
Fully aware of new media technology	40
Neutral	15
Total	100

### Understanding New Media Technology

From Table 3, it can be deduced that of all participants, 40% of them reported that they were fully aware of the term and concept of new media technology, 30% of them reported that they were partially aware of the term and concept of new media technology, 15% of them reported that they were unaware of term and concept of new media technology, and another 15% of them stayed neutral.

### Characteristics of New Media Technologies

From Table 4, it can be inferred that out of 100 participants, communication features cater to 30%, convergence and community cater to 10%, the creativity of the users cater to 30%, and collaboration caters to 30%, which will positively affect the behavior of an individual/user in this digital age. This shows that almost all characteristics of new media technology impact the behavior of an individual.

**Table 4:** Characteristics of new media technologies.

**Characteristics of new media technologies**	**Respondents' response in percentage (%)**
Communication	30
Convergence and community	10
Creativity	30
Collaboration	30
Total	100

### New Media Technologies and Digital Age

From Table 5, it can be inferred that out of 100 participants 70% of them agreed that there is a positive relationship between new media technology and the digital age, while 20% of them agreed that there is a negative relationship between new media technology and the digital age. often percent stayed neutral. This shows that there certainly exists a relationship between new media and the digital age.

**Table 5:** New media technology and digital age.

**New media technologies and digital age**	**Respondents' responses by percentage (%)**
Positive relationship	70
Negative relationship	20
Neutral	10
Total	100

### Mediating Role of Engagement and Interactivity

From Table 6, it can be inferred that out of 100 participants 80% of them agreed that new media plays a mediating or engaging role in this digital age to share information, view content, post feedback on blogs, and so on. Meanwhile 15% of them agreed that interactivity is essential in new media technologies, especially in this digital age, and hence interactivity plays a mediating role. Only 5% of the respondents stayed neutral. This shows that both engagement and interactivity play a mediating role between new media and the digital age. New media engagement is essential to share information with each other, develop creative content, create branding, and so on.

**Table 6:** Mediating role of engagement and interactivity.

**Mediating role of engagement and interactivity**	**Respondents' responses by percentage (%)**
New media engagement	80
New media interactivity	15
Neutral	5
Total	100

### Results of Rating Scale

**Figure 4:**
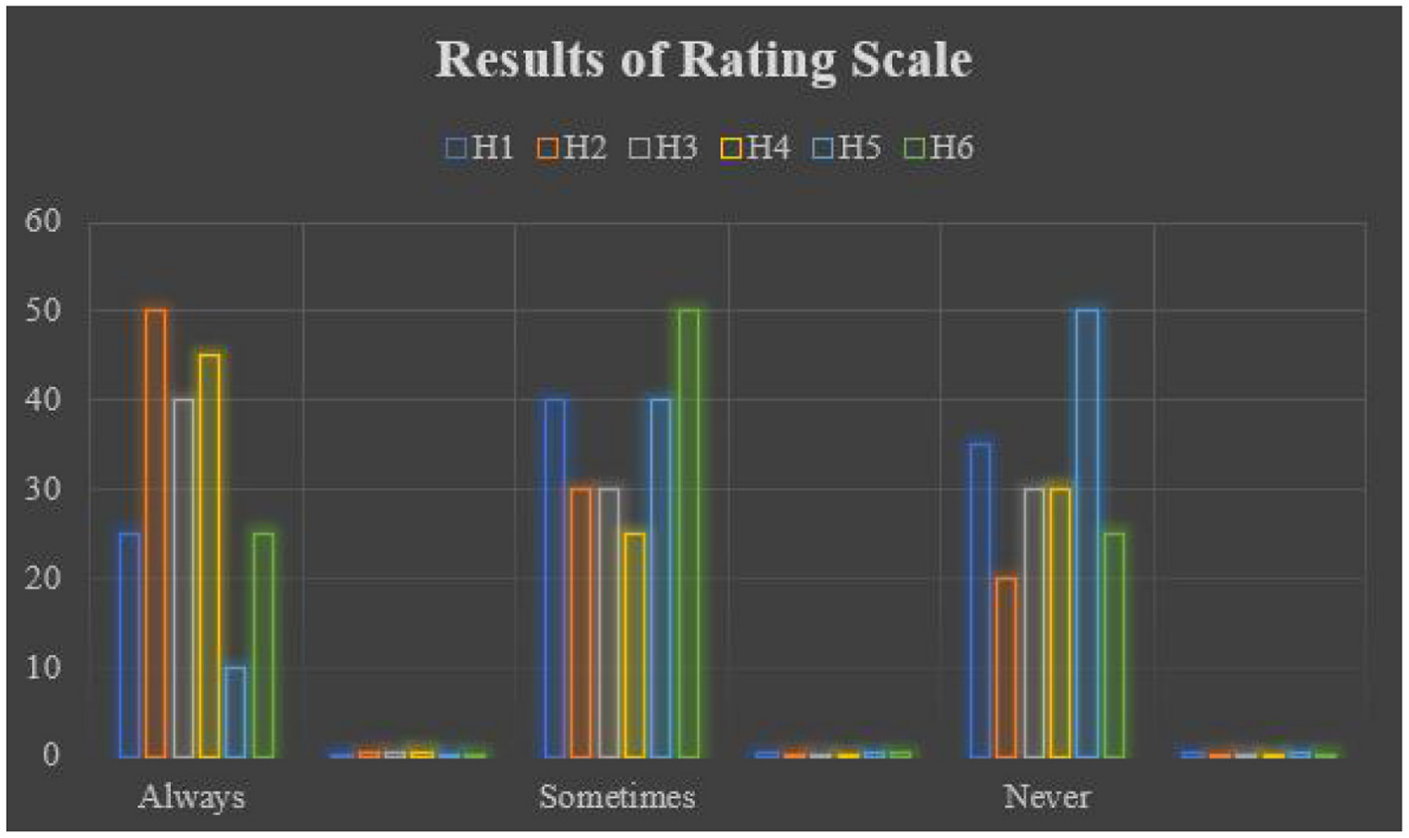
Graphical representation of rating scale results.

Table 7 records the statistical analysis of the rating scale selected in this study. The results of the rating scale were analyzed on a three-point Likert scale ranging from “Always” to “Never.” Figure 4 presents the graphical representation of the results of the rating scale.

**Table 7:** Results of rating scale.

**Rating Scale**	**Respondents' response rate**						**Total**	
	**Always**		**Sometimes**		**Never**		
	***R***	**%**	***R***	**%**	***R***	**%**	***R***	**%**
Most of the users are aware of new media technologies	25	25	40	40	35	35	100	100
The characteristics of new media technologies positively influence the user behavior	50	50	30	30	20	20	100	100
There is a relationship between new media technologies and digital age	40	40	30	30	30	30	100	100
Almost all the people in this digital age use web-technologies for communication	45	45	25	25	30	30	100	100
New media technologies enables interactivity among users	10	10	40	40	50	50	100	100
New media plays a mediating role in this digital age	25	25	50	50	25	25	100	100

The findings of the current study are given in the given Table 8 given below.

**Table 8:** Item statics.

**Items**	**Levels**	
	**M**	**MI**
H1	5.23	5.29
H2	5.13	5.30
H3	4.69	5.20
H4	5.00	5.91
H5	5.19	5.90
H6	5.41	5.72

*M, mean performance; MI, Mean Importance; SD, standard deviation*.

**Figure 5:**
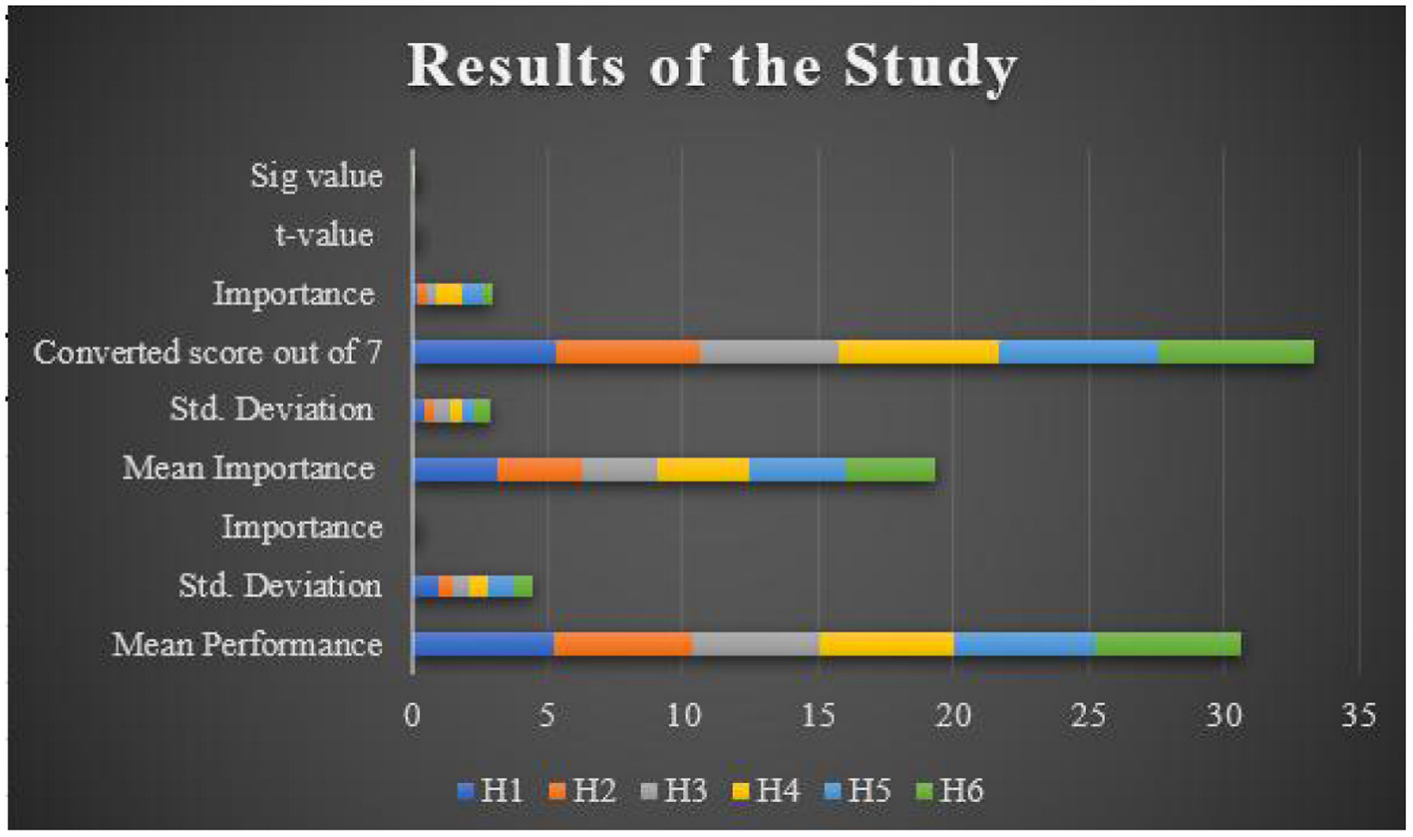
Assessing the measures.

Figure 5 shows the assessing the measures for different parameters. The current study analyzed the mediating role of engagement between new media and information in this digital age. The demographic data of respondents were also considered, which helped us to understand if the respondents were aware of the new media technologies that are rapidly spreading in this digital age. The results suggest that people in the digital age are aware of the new media web-communication technologies. Moreover, the results also indicate that almost 90% of the respondents think that communication convergence, community, creativity, and collaboration are the five major characteristics of new media, which influences the use of new media in this twenty first century. In addition to, these characteristics are generalized and can be applied worldwide, as everything has become online these days. Furthermore, the study also found that there is a positive relationship between new media technology and the digital age. A digital age permits the users to adopt different new media web-communication technologies in their daily life for easier and smooth communication. Results of the study stated that new media plays a mediating or engaging role in this digital age to share information, view content, post feedback on blogs and so on. The study also found that interactivity is essential in new media technologies, especially in this digital age, and hence interactivity plays a mediating role. Both engagement and interactivity play a mediating role between new media and digital age. New media engagement is essential to share information with each other, develop creative content, create branding, and so on. The current study can be seen as a beginning and future researchers can explore mediating role of other factors in the digital age. It is also suggested to extend the number of respondents for more information on this study. User engagement plays an important role in transmitting the message to the target audience. Likewise, it is also traced that most of the people in this digital age posts activities through various social media platforms. In short, it can be stated that user participation is very important to process any kind of information. These new media technologies are changing the behavior of the people, enabling them to interact online, create online communities, and become socially engaging. Thus, it can be argued that engagement of the audience/users/individuals is crucial in new media technology communication. Table 9 shows the performance measure as shown below.

**Table 9:** Assessing the measures.

**Performance**	**Mean performance**	**Std. deviation**	**Importance**	**Mean importance**	**Std. deviation**	**Converted score out of 7**	**Importance**	***t*-value**	**Sig value**
H1	5.23	0.98	H1	3.14	0.41	5.29	0.20	−2.785	0.015
H2	5.13	0.50	H2	3.12	0.38	5.30	0.3	−3.214	0.014
H3	4.69	0.60	H3	2.82	0.59	5.20	0.39	−3.764	0
H4	5.00	0.69	H4	3.41	0.42	5.91	0.91	−12.512	0
H5	5.19	0.99	H5	3.52	0.51	5.90	0.85	−10.792	0
H6	5.41	0.71	H6	3.35	0.61	5.72	0.3	−2.412	0.022

## Conclusion

The present article has proposed and tested the mediating role of new media engagement in this digital age. Interactivity is essential in new media technologies, especially in this digital age, and hence interactivity plays a mediating role. Both engagement and interactivity play a mediating role between new media and the digital age. New media engagement is essential to share information with each other, develop creative content, create branding, and so on. This research analyzed the characteristics of new media technologies which affect the communication process in all organizations across various fields. The results of the study indicated that new media technologies influence the behavior of the people, enabling them to interact online, create online communities, and become socially engaging. Thus, it can be argued that engagement of the audience/users/individuals is crucial in new media technology communication. Future researchers can adopt a scientific approach to understand the mediating role of new media engagement in this digital age. They can focus on the element of interactivity by conducting case studies to have much more appropriate results that can apply across the fields.

## Data Availability Statement

The original contributions presented in the study are included in the article/supplementary material, further inquiries can be directed to the corresponding author/s.

## Author Contributions

All authors listed have made a substantial, direct, and intellectual contribution to the work and approved it for publication.

## Conflict of Interest

The authors declare that the research was conducted in the absence of any commercial or financial relationships that could be construed as a potential conflict of interest.

## Publisher's Note

All claims expressed in this article are solely those of the authors and do not necessarily represent those of their affiliated organizations, or those of the publisher, the editors and the reviewers. Any product that may be evaluated in this article, or claim that may be made by its manufacturer, is not guaranteed or endorsed by the publisher.
